# A combined therapeutic approach: extracorporeal shock wave therapy and botulinum toxin in multiple sclerosis-related spasticity

**DOI:** 10.3389/fneur.2026.1851860

**Published:** 2026-06-16

**Authors:** Arianna Sartori, Anna Favero, Laura Mazzari, Giulia Mazzon, Alessio Bratina, Antonio Bosco, Marinella Tomaselli, Paolo Manganotti

**Affiliations:** 1 Neurology Unit, Hospital Care Department of Medicine, Azienda Sanitaria Universitaria Giuliano Isontina (ASUGI), Trieste, Italy; 2 Neurology Unit, Department of Medical, Surgical and Health Sciences, University of Trieste, Trieste, Italy

**Keywords:** botulinum toxin injection, focal extracorporeal shock wave therapy, multiple sclerosis, spasticity, symptomatic treatment

## Abstract

**Introduction:**

Focal extracorporeal shock wave therapy (fESWT) is a non-invasive treatment for spasticity in neurological disorders; evidence in multiple sclerosis (MS) is limited. The aim of this study is to evaluate whether its combination with botulinum toxin injections (BTI) could provide rapid and sustained clinical benefit.

**Methods:**

This single-center retrospective study included people with MS-related spasticity treated with fESWT and BTI. Global perceived benefit was collected. Modified Ashworth Scale (mAS), Penn Spasm Frequency Scale (frequency—fPSFS and intensity—iPSFS), Numeric Rating Scale for pain (NRS) were collected at baseline (pre and post-treatment), at day 7 and 28. To assess the treatment efficacy on all muscle groups, linear mixed-effects models were implemented. As exploratory analyses, a general linear model for repeated measures was applied for the subgroup analysis aimed at evaluating the efficacy of the treatment in each muscle group.

**Results:**

Fifteen PwMS were enrolled. The treated muscles were: adductors, rectus femoris, hamstrings, triceps surae, and biceps brachii. Fourteen subjects (93.3%) reported an immediate benefit, confirmed 28 days later. Eight subjects (53.3%) reported sustained benefit in spasticity. In all-muscles analysis, significant effect was found for mAS [*F*(3,161.139) = 6.799, *p* < 0.001], and iPSFS [*F*(3,162.408) = 3.619, *p* = 0.014]. In muscle-groups analysis, a significant effect on mAS was observed in adductors [*F*(3,27) = 20.335; *p* < 0.001], rectus femoris *F*(3,18) = 11.547; *p* < 0.001, hamstrings [*F*(3,12) = 5.253; *p* = 0.015] and triceps surae [*F*(1.536, 7.682) = 5.636; *p* = 0.037].

**Discussion:**

This study suggests that combined treatment with fESWT+BTI is effective for the treatment of MS-related spasticity, with fESWT offering non-invasive and immediate relief while botulinum toxin becomes fully effective.

## 1 Introduction

Spasticity is defined as “*a motor disorder that is characterized by a velocity-dependent increase in tonic stretch reflexes (muscle tone) with exaggerated tendon jerks, resulting from hyperexcitability of the stretch reflex as a component of the upper motor neuron syndrome*” (1). In multiple sclerosis (MS), spasticity is among the most frequent and functionally limiting clinical features (2). It reflects involvement of the upper motor neuron pathway and originates from early demyelinating and axonal injury (3). The consequent disruption of descending inhibitory control over spinal reflex circuits produces abnormal muscle overactivity, affecting the trunk and extremities, particularly the lower limbs (4). Spasticity affects approximately 65% of patients with MS, and about one third of patients experience it at least at a moderate level (5). Spasms, characterized as complex, involuntary motor phenomena frequently elicited by sensory or auditory stimuli and often accompanied by pain, represent a core clinical feature within the spectrum of spasticity, particularly in patients with MS (6). Collectively, the symptoms (pain, allodynia, bladder dysfunction, fatigue, and sleep disturbances) that accompany spasticity in patients with MS are also known as *spasticity plus syndrome* (7).

The management of spasticity encompasses a range of therapeutic strategies, including physiotherapy interventions and pharmacological treatments such as benzodiazepines, cannabinoids (8), baclofen (administered orally or intrathecally) and botulinum toxin type A (9, 10). However, these therapeutic approaches may be associated with long-term side effects that limit their sustained use, can contribute to the development of tolerance and, particularly in the case of botulinum toxin, may require progressively increasing doses over time.

Low-energy extracorporeal shock wave therapy (ESWT) has recently emerged as a non-invasive neurological intervention primarily employed to reduce spasticity and hypertonia in conditions such as stroke, cerebral palsy, and MS (11–16). Evidence from both preclinical and clinical studies indicates that ESWT may transiently attenuate muscle hypertonia via mechanisms such as reduced acetylcholine release at the neuromuscular junction, increased nitric oxide (NO) production, and anti-inflammatory effects (17, 18). To date, the literature regarding the use of ESWT in MS remains extremely limited. Radial shock wave therapy (RSWT) as a stand-alone treatment was investigated in a single randomized, double-blind, placebo-controlled study involving 34 people with MS (PwMS), demonstrating efficacy in reducing pain and muscle tone (19). ESWT has also been investigated as an adjunctive treatment to botulinum toxin injections (BTI) in two studies (12, 20), both suggesting that this combination may prolong therapeutic efficacy compared with BTI alone. Finally, one study compared focal *vs.* unfocused shock waves combined with BTI in patients with both MS and stroke (11). These findings suggest that integrating these approaches into spasticity management protocols could offer significant advantages for PwMS. To our knowledge, no studies have investigated the immediate effects of fESWT administered in combination with BTI.

The aim of this study is to evaluate the effectiveness of combining fESWT with the immediate subsequent administration of botulinum toxin for the treatment of spasticity in PwMS. In particular, the objective is to determine whether fESWT+BTI can help provide a rapid and sustained therapeutic effect on spasticity, spasms and pain in the population, with particular attention on the timing of benefit onset, since botulinum toxin treatment alone generally requires 7–14 days to achieve its full clinical effect.

## 2 Materials and methods

This observational retrospective single-center study was conducted on PwMS suffering from spasticity followed in our Spasticity Outpatient Center (Neurology Unit, University of Trieste), treated with a combination of BTI, in particular incobotulinumtoxinA (INCO, Xeomin®) and fESWT from March 2025 to January 2026. Inclusion criteria were: 1) diagnosis of MS; 2) treatment with fESWT and BTI; 3) availability of clinical data of neurological follow-up at 7 and 28 days after treatment. All participants provided written informed consent in accordance with the Declaration of Helsinki. The study was approved by the local University local ethics committee.

### 2.1 fESWT

An electromagnetic coil lithotripter (Duolith Storz Medical AG) was used. The intervention was performed using fESWT, applied in the same muscles where botulinum was injected. Each patient received from 500 to 1,500 impulses per muscle, with the best tolerated level of energy [from 0.15 to 0.40 mJ/mm (2)] and a frequency of 4 Hz over the area of the muscles affected. The procedure is non-invasive, usually painless and does not require anesthesia or analgesics.

### 2.2 BTI

Patients received treatment with INCO. Dosages were measured in units (U), with a dilution ratio of 1:2. All injections were administered by two experienced physicians, primarily under ultrasound guidance, with electromyography guidance applied in selected cases. The selection of target muscles and the administered dose were individualized based on therapeutic objectives, clinician-evaluated spasticity severity, and the patients’ own perception of their spasticity.

### 2.3 Clinical variables

For each participant we collected the following clinical data: sex, age, disease duration, disease type, disability evaluated with Expanded Disability Status Scale (EDSS), type of spasticity, concomitant symptomatic treatment for spasticity, concomitant disease modifying drugs (DMDs). Treatment related variables were considered, in particular: BTI dose (U), and for fESWT number of impulses, energy and frequency.

Data regarding self-reported spasticity improvement were collected, both as general benefit and as a muscle-specific evaluation. Onset and duration of benefit were recorded.

Modified Ashworth Scale (mAS), Penn Spasm Frequency Scale (in terms of frequency—fPSFS and intensity—iPSFS) and Numeric pain Rating Scale (NRS) were collected at baseline—before (T0-pre) and after (T0-post) treatment (evaluated 30 min after combined treatment), day 7 (T1) and day 28 (T2) after treatment.

### 2.4 Outcomes

The primary outcomes were:

- The proportion of subjects reporting an immediate perceived benefit on spasticity, evaluated 30 minutes after the combined treatment, and a sustained benefit at 4 weeks;
- The duration of the perceived benefit.

The secondary outcomes were:

- The proportion of treated muscles/muscle groups in which a benefit was perceived at the four assessment time points;
- The description of treated muscle groups and the comparison of treatment-related parameters among the different muscle groups;
- The efficacy of the combined treatment (fESWT+BTI) on mAS, fPSFS, iPSFS, and NRS scores across all treated muscles at the four time points.

The exploratory outcome was:

- The efficacy of the combined treatment (fESWT+BTI) according to muscle/muscle group and individual patient analyses.

### 2.5 Statistical analysis

Shapiro–Wilk test was used to assess the normality of the distribution of continuous data. Continuous variables were presented as median (range) or mean ± standard deviation (SD), according to distribution.

To compare treatment related variables across muscle/muscle groups (BTI dosage, number of impulses, and fESWT energy for each muscle group) linear mixed-effects models were applied to account for the hierarchical structure of the data, with repeated observations nested within patients. Muscle group was included as a fixed effect in all models, while patient identity was modelled as a random intercept to account for inter-individual variability. Separate models were fitted for each dependent variable. Pairwise comparisons between muscle groups were performed using Bonferroni correction to adjust for multiple testing. The frequency of fESWT was kept constant across all muscles and subjects and was therefore not included in the analyses.

To examine the effect of time on mAS, PSFS, iPSFS, and NRS scores while accounting for the hierarchical structure of the data, linear mixed-effects models were implemented. Time was included as a fixed effect with four levels (T0-pre, T0-post, T1, T2), with T2 serving as the reference category. Muscle type was included as an additional fixed factor to account for between-muscle differences. The interaction between time and muscle type was also initially considered. Random effects were specified at the patient level, with random intercepts included to account for inter-individual variability in baseline spasticity, spasms and pain variables. Pairwise comparisons of estimated marginal means were performed with Bonferroni adjustment to control for multiple comparisons.

Finally, as an exploratory analysis, in order to assess the effect of the combined treatment on mAS, fPSFS, iPSFS, and NRS, for each muscle/muscle group separately, a general linear model (GLM) for repeated measures was applied, considering time as a within-subject factor. Bonferroni correction was used for pairwise comparisons. If subjects were treated bilaterally in the same muscle/muscle group, the mean value was used for analysis.

Statistical analyses were performed using IBM SPSS Statistics software, version 31.0. A *p*-value ≤0.05 was considered statistically significant.

## 3 Results

### 3.1 Subjects characteristics and treated muscles

**Figure 1:**
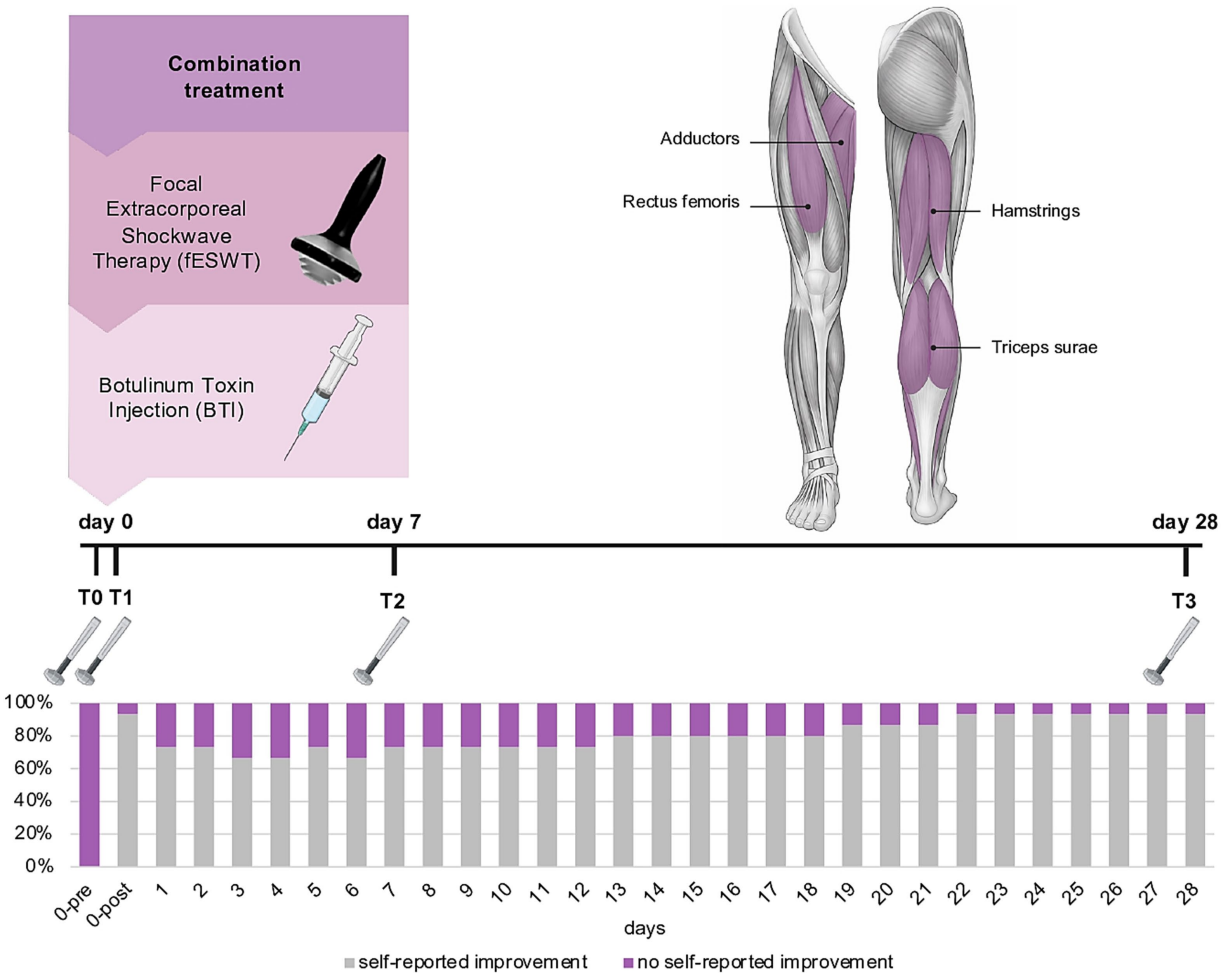
Lower limb treated muscles and self-reported improvement of combined treatment according to timeline [created in BioRender. Sartori, A. (2026) https://BioRender.com/0j200w0].

Data were collected from 15 PwMS. Baseline demographic and clinical characteristics of PwMS are summarized in Table 1. The treated muscles/muscle groups included adductors, rectus femoris, hamstrings, triceps surae, and biceps brachii (Figure 1). All treated patients reported spasticity at baseline, whereas only 3 patients (20%) reported pain.

**Table 1:** Patients’ demographic and clinical characteristics at baseline.

Clinical characteristics	*n* = 15
Sex, F, *n* (%)	8 (53.3)
Age, mean ± SD	64.4 ± 9.7
Disease duration (years), mean ± SD	24.3 ± 8.4
EDSS, median (range)	7 (3–8)
Disease form, *n* (%)	
Relapsing remitting	2 (13.3)
Secondary progressive	7 (46.7)
Primary progressive	6 (40.0)
Type of spasticity, *n* (%)	
Focal	1 (6.7)
Paraspasticity	12 (80.0)
Tetraspasticity	2 (13.3)
Previous/concomitant pharmacological treatments for spasticity, *n* (%)	
N treated patients, *n* (%)	13 (86.7)
N treatments, median (range)	1 (0–3)
Type of treatments for spasticity, *n* (%)	
Baclofen	11 (73.3)
Benzodiazepines	5 (33.3)
Cannabinoids	5 (33.3)
Previous/concomitant physiotherapy, *n* (%)	11 (73.3)
Concomitant DMDs, *n* (%)	
First line	2 (13.3)
Second line	4 (26.7)
None	9 (60.0)
Botulinum toxin	
Type: incobotulinumtoxinA, *n* (%)	15 (100)
Dose: units, mean ± SD	175.0 ± 72.6
Patients previously treated with BTI, *n* (%)	14 (93.3)
Botulinum toxin treatment duration (days), mean ± SD	67.3 ± 38.7
N of patients treated with both fESWT and BTI, *n* (%), bilaterally *n* (%)	
Adductors	10 (66.7)
Rectus femoris	7 (46.7)
Hamstrings	5 (33.3)
Triceps surae	6 (40)
Biceps brachii	1 (6.7)
N muscle treated per patient, median (range)	3 (1–4)

SD, standard deviation; EDSS, expanded disability status scale; DMDs, disease modifying drugs; fESWT, focal extracorporeal shockwave treatment; BTI, botulinum toxin injection.

### 3.2 Subjects’ perceived benefit: onset, duration, and percentage of muscles with reported improvement

Fourteen subjects (93.3%) reported an immediate subjective improvement in hypertonia following the combined treatment and the same benefit was also observed at 4-week follow-up. Among these patients, the combined (fESWT+BTI) treatment led to a mean duration of clinical benefit of 130.2 ± 27.2 days. Eight subjects (53.3%) experienced continuous benefit, with no interval between the end of the fESWT effect and the onset of the BTI effect. In the remaining six subjects, the mean gap in effectiveness between the two treatments was 9 ± 5.8 days; fESWT showed a mean duration of effect of 2.7 ± 2.1 days, and an average delay of 11.7 ± 7.6 days in the onset of clinical benefit from botulinum toxin.

The effectiveness of the combined treatment at individual treatment sites was also assessed, based on patient-reported outcomes and clinical characteristics collected at four time points. Sixty percent of subjects experienced benefit in 100% of the treated muscles immediately after the combined treatment. This percentage increased to 73.3% at both day 7 and day 28 (Supplementary Figure S1).

### 3.3 Comparison of treatment-related parameters among treated muscle groups

Treatment parameters are summarized in Table 2, including botulinum toxin dosage, number of impulses, fESWT energy and frequency for each muscle group.

**Table 2:** Parameters of the treatments (fESWT+BTI) according to muscle/muscle group.

Treated muscles (*n* = 44)	Adductors	Rectus femoris	Hamstrings	Triceps surae	Biceps brachii
*N*, (%)	16 (36.4)	13 (29.5)	6 (13.6)	7 (15.9)	2 (4.5)
N treated patients, *n* (%)	10 (66.7)	7 (46.7)	5 (33.3)	6 (40)	1 (6.7)
Bilateral treatment, *n* (%)	6 (60)	6 (85.7)	1 (20)	1 (16.7)	1 (100)
BTI dosage, median (range)	100 (30–100)	40 (10–100)	35 (10–100)	50 (50–125)	50 (50–50)
fESWT					
Hits (Sb), median (range)	750 (700–1,500)	750 (700–1,500)	1,450 (1400–1,500)	1,000 (500–1,500)	750 (750–750)
Energy (mJ/mm2), median (range)	0.40 (0.35–0.50)	0.40 (0.35–0.50)	0.40 (0.40–0.40)	0.40 (0.15–0.40)	0.40 (0.40–0.40)
Frequency (Hz), median (range)	4 (4–4)	4 (4–4)	4 (4–4)	4 (4–4)	4 (4–4)
Pain during fESWT, *n* (%)	0 (0)	0 (0)	2 (33.3%)	0 (0)	0 (0)
Pain after fESWT, *n* (%)	0 (0)	0 (0)	2 (33.3%)	0 (0)	0 (0)

BTI, botulinum toxin injection; SD, standard deviation; fESWT, focal extracorporeal shockwave treatment.

A linear mixed-effects model was used to assess differences in botulinum toxin dosage across muscle groups, accounting for clustering within patients. A significant main effect of muscle group was found [*F*(4,39) = 4.317, *p* = 0.005]. The model explained 30.7% of the variance (marginal *R^2^*=0.307). Post-hoc Bonferroni-adjusted comparisons indicated that adductor received significantly higher doses than rectus femoris (*p* = 0.007).

The analysis was repeated for the number of fESWT impulses (hits); a statistically significant effect of muscle group was observed [*F*(4,39) = 3.553, *p* = 0.015]. The model explained 26.7% of the variance (marginal *R^2^* = 0.267). According to post-hoc Bonferroni-adjusted pairwise comparisons, hamstrings received higher number of impulses compared to rectus femoris (*p* = 0.010).

Finally, the linear mixed-effects model for fESWT energy intensity showed again a significant effect of muscle group [*F*(4,39) = 3.084, *p* = 0.027]. The model explained 24.0% of the variance (marginal *R^2^* = 0.240). Post-hoc pairwise comparisons with Bonferroni correction revealed significant differences between triceps surae and both adductors (*p* = 0.031) and rectus femoris (*p* = 0.025).

No other pairwise comparisons reached statistical significance. Estimated marginal means are reported in Supplementary Table S1.

Overall, these findings indicate that BTI dose, number of fESWT impulses and fESWT energy intensity differed significantly across the treated muscle groups. The very small number of observations for biceps brachii (*n* = 2) resulted in wide confidence intervals.

Finally, it is noteworthy that no pain was reported either during or after the treatment, except in two cases involving the hamstrings.

### 3.4 Efficacy of the combined treatment in all treated muscles across the four time points

**Figure 2:**
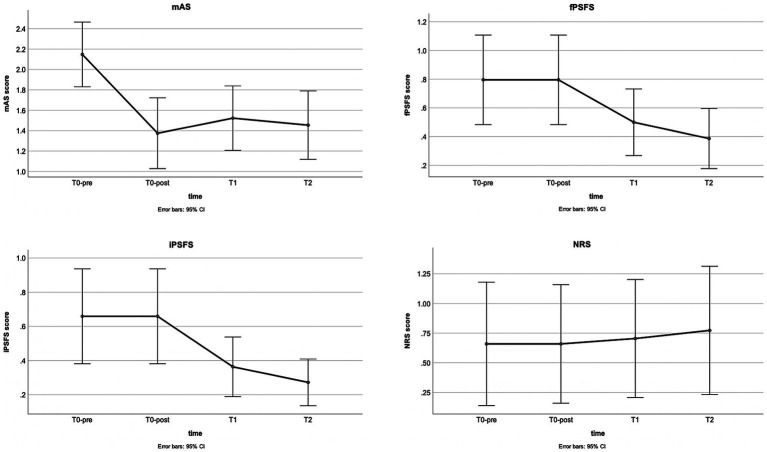
MAS, fPSFS, iPSFS, and NRS across the four time points (in all muscles/muscle groups).

Effectiveness of the combined (fESWT+BTI) treatment was analyzed in all treated muscles, and mAS, fPSFS, iPSFS, and NRS scores were compared across the four time points (T0-pre, T0-post, T1, and T2; Figure 2) using linear mixed-effects models. The biceps muscle was excluded from the analysis because of the very limited number of observations (*n* = 2, one patient) and zero within-subject variability in all scores.

A significant effect of time on mAS scores was observed [*F*(3,161.139) = 6.799, *p* < 0.001]. Pairwise comparisons indicated that baseline (T0-pre) values were significantly higher than all subsequent time points (T0-post mean difference = 0.810, 95%CI 0.273, 1.346, *p* < 0.001; T1 = 0.655, 95%CI 0.118, 1.192, *p* = 0.008; T2 = 0.726, 95%CI 0.189, 1.263, *p* = 0.002), whereas no significant differences were observed among post-treatment measurements (T0-post, T1, T2), suggesting an early reduction in spasticity followed by a stable plateau over time. The model explained 33.0% of the variance.

For fPSFS scores, the analysis did not reveal a statistically significant effect of time (*F*(3,162.502) = 2.490, *p* = 0.062). Although a decreasing trend was observed across time points, pairwise comparisons with Bonferroni correction did not show any statistically significant differences between time points.

The linear mixed-effects model showed a statistically significant effect of time on iPSFS scores [*F*(3,162.408) = 3.619, *p* = 0.014]. Estimated marginal means indicated a progressive reduction in iPSFS scores over time, with no change between baseline and immediate post-treatment (T0-pre = 0.69, 95%CI 0.61, 0.77; T0-post = 0.69, 95%CI 0.61, 0.77), followed by a gradual decrease at T1 (0.38, 95%CI 0.30, 0.46) and T2 (0.26, 95%CI 0.18, 0.34). However, pairwise comparisons with Bonferroni correction did not reveal statistically significant differences between individual time points, although a borderline trend was observed between baseline and T2 (mean difference = 0.429, 95%CI −0.005, 0.863, *p* = 0.055).

No significant effect of time on NRS scores was observed [*F*(3,161.432) = 0.069, *p* = 0.976]. All pairwise comparisons between time points were non-significant, confirming the absence of detectable differences over time.

### 3.5 Efficacy of the combined treatment according to muscle/muscle group and patient across the four time points

As exploratory outcome, we compared the effectiveness of the combined (fESWT+BTI) treatment by muscle/muscle group. Subjects were analyzed individually. In cases of bilateral combined treatment on the same muscle/muscle group, the mean value was used for analysis (the analysis was also repeated considering the more severely affected side, yielding the same results). Treatment details are reported in Supplementary Table S2. Scores for mAS, fPSFS, iPSFS, and NRS at the different time points are presented in Supplementary Table S3.

**Figure 3:**
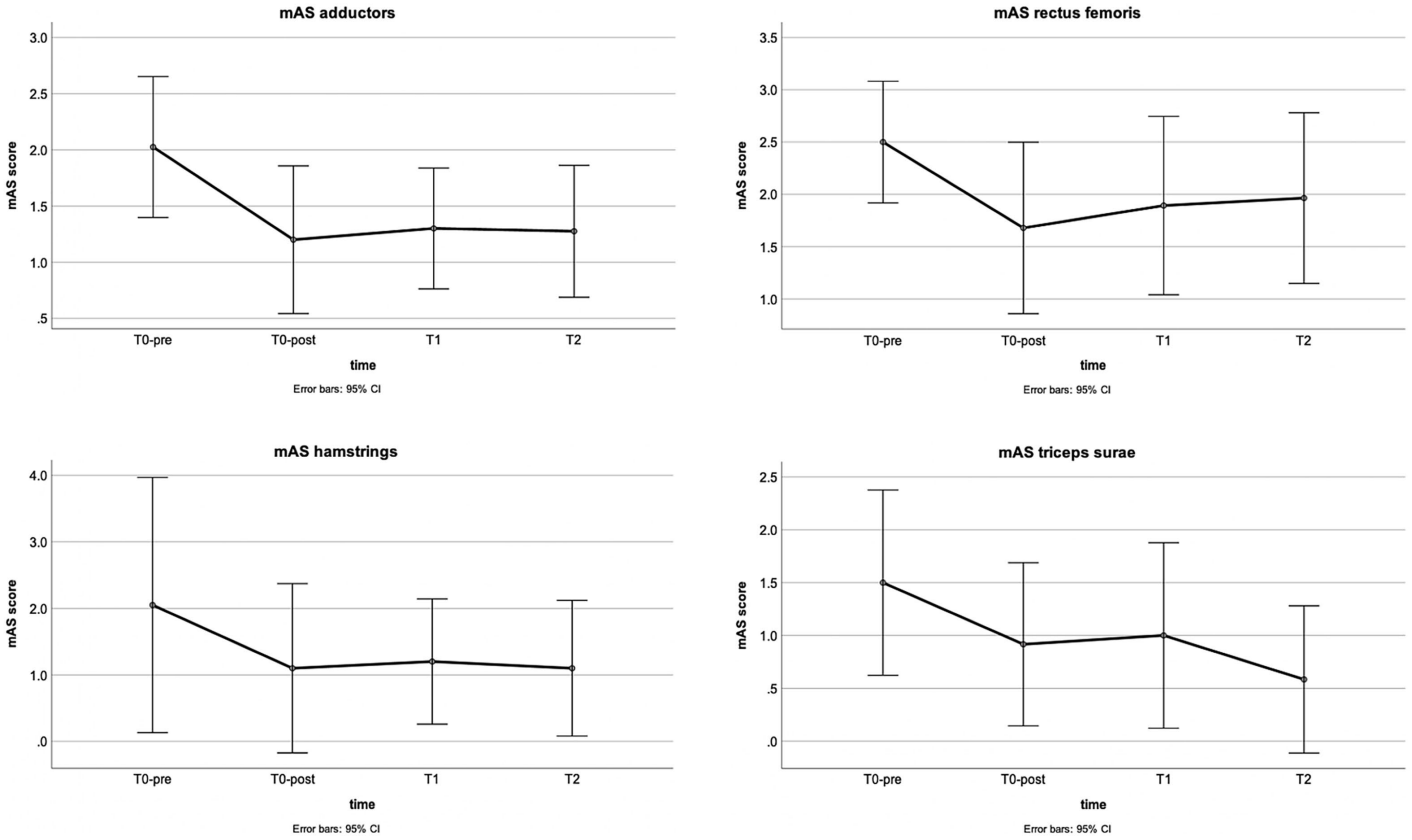
MAS scores across the four time points for each muscle/muscle group.

For mAS, the repeated-measures GLM showed the following results for each muscle group (Table 3, Figure 3):

- *Adductors*: significant effect of time [*F*(3,27) = 20.335; *p* < 0.001; partial η^2^ = 0.693]. In particular, a statistically significant difference was observed between T0-pre and T0-post, T1, and T2, with a reduction in scores maintained over time.
- *Rectus femoris*: significant effect of time [*F*(3,18) = 11.547; *p* < 0.001; partial η^2^ = 0.658]. Specifically, a statistically significant difference was observed between T0-pre and T0-post.
- *Hamstrings*: significant effect of time [*F*(3,12) = 5.253; *p* = 0.015; partial η^2^ = 0.568]. However, pairwise comparisons with Bonferroni correction did not show statistically significant differences.
- *Triceps surae*: significant effect of time [*F*(1.536, 7.682) = 5.636; *p* = 0.037; partial η^2^ = 0.530]. However, pairwise comparisons with Bonferroni correction did not reveal statistically significant differences.

**Table 3:** Comparison of mAS across the four time points.

Muscles	T0-pre vs T0-post		T0-pre vs T1		T0-pre vs T2		T0-post vs T1		T0-post vs T2		T1 vs T2	
	Mean diff (CI 95%)	*p*	Mean diff (CI 95%)	*p*	Mean diff (CI 95%)	*p*	Mean diff (CI 95%)	*p*	Mean diff (CI 95%)	*p*	Mean diff CI 95%)	*p*
Adductors (*n* = 16)	0.825 (0.339–1.311)	**0.009**	0.725 (0.232–1.218)	**0.015**	0.750 (0.298–1.202)	**0.013**	−0.100 (−0.520–0.320)	1.000	−0.075 (−0.383–0.233)	1.000	0.025 (−0.171–0.221)	1.000
Rectus femoris (*n* = 15)	0.821 (0.132–1.511)	**0.022**	0.607 (−0.055–1.269)	0.073	0.536 (−0.039–1.110)	0.068	−0.214 (−0.789–0.360)	1.000	−0.286 (−0.820–0.249)	0.073	−0.071 (−0.250–0.107)	1.000
Hamstrings (*n* = 4)	0.950 (−0.695–2.595)	0.292	0.850 (−1.013–2.713)	0.548	0.950 (−0.782–2.681)	0.338	−0.100 (−1.288–1.088)	1.000	0.0 (−0.767–0.767)	1.000	0.100 (−0.585–0.385)	1.000
Triceps surae (*n* = 10)	0.583 (−0.065–1.232)	0.076	0.500 (−0.270–1.270)	0.245	0.917 (−0.228–2.061)	0.118	−0.083 (−0.435–0.268)	1.000	0.333 (−0.710–1.376)	1.000	0.417 (−0.963–1.796)	1.000

Bold values indicate statistical significance.

No significant effects were found for fPSFS, iPSFS, or NRS in any muscle group (data not shown) (Supplementary Figures S2–S4).

Since only one subject was treated at the level of the biceps brachii (with no evidence of significant improvement), no formal analysis was performed for this muscle.

## 4 Discussion

Our study demonstrates that combining fESWT and BTI is effective in treating focal spasticity in MS. We observed a significant improvement over time (at 7 and 28 days) in both spasticity (mAS) and, to a lesser degree, in spasms intensity when all treated muscles were considered collectively. No effect was observed on spasm frequency or pain outcome. According to the exploratory analysis based on stratification by muscle group, the effect on spasticity was confirmed for the adductors and rectus femoris. However, statistical significance was not reached for the other lower limb muscle groups, likely due to the small sample size, although the overall trend remained consistent.

Our findings support the effectiveness of the combined (fESWT+BTI) treatment, in accordance with the three available studies in the literature (11, 12, 20). Marinaro and colleagues treated 16 PwMS with spasticity of triceps surae muscles using BTI followed, 4 months later, by four sessions of radial ESWT (rESWT). The authors reported that rESWT appeared to prolong the BTI-mediated reduction in spasticity and improve both passive and active ankle range of motion (20). Déniz and colleagues compared the efficacy of focal *vs.* unfocused shock waves combined with BTI in MS and stroke patients, reporting improvements in mAS scores at 5, 12, and 25 weeks after treatment in both intervention groups and across both disease populations (11). Finally, the same group investigated the efficacy of fESWT+BTI in MS and stroke; eight PwMS were included and initially received BTI alone, followed by combined treatment consisting of BTI + fESWT and three additional weekly fESWT sessions. The authors observed a significantly greater improvement in mAS scores after the combined treatment compared with BTI alone (12). In that study, the first post-treatment evaluation was performed 1 week after the intervention. To date, no studies have specifically investigated the immediate effects of fESWT. Our study differs from previous reports in terms of target population (only PwMS were included), treatment protocol (a single fESWT session administered immediately before BTI), and timing of efficacy assessments (T0: 30 min after treatment; T1: 1 week after treatment; T2: one month after treatment). Despite these methodological differences, our findings further support the effectiveness of the combined treatment in reducing spasticity, although direct comparisons among studies remain difficult. The rationale for combining the two treatments through this approach is based on the hypothesis that ESWT can favorably modify the muscular microenvironment targeted by botulinum toxin. Specifically, fESWT may exert anti-inflammatory effects by increasing nitric oxide production and modulating oxidative stress, thereby reducing chronic inflammation (18); additionally, it may facilitate mechanotransduction through activation of signaling pathways and the release of biomolecules, ultimately contributing to pain relief (21). We hypothesize that the lack of effect on pain relief in our study could have been related to the limited number of patients reporting pain at baseline (20%).

The most notable finding of the combined (fESWT+BTI) treatment is the effect on spasticity not only over time but also immediately after treatment. We hypothesized that the rapid improvement in hypertonia observed after treatment may reflect a contribution of fESWT, which may act as a temporary “bridge,” helping patients maintain functional improvements until botulinum toxin becomes fully effective (7–14 days) (22). This is further supported by the high percentage (>53%) of patients reporting continuous overall improvement, with either no interval or only a short mean interval (9 days) between the effect of the two treatments. Taken together, these observations support the potential usefulness of a combined treatment strategy in the management of focal spasticity in PwMS, in which initial fESWT may help to prime or modulate the targeted muscles, followed by BTI to consolidate and prolong the therapeutic effect.

Regarding patient characteristics, the mean age and disability scores were relatively high, as expected in a population where spasticity becomes particularly relevant and requires second or third-line treatments (23). Indeed, the vast majority of subjects had already unsuccessfully tried at least one oral treatment for MS-related spasticity (24). Similarly, more than 80% of patients had a progressive form of the disease, which explains why only 40% were receiving disease-modifying therapies.

The most frequently treated muscle groups were the adductors, rectus femoris, triceps surae, and hamstrings. Only one patient was treated in the upper limb, specifically targeting the biceps brachii. These findings are consistent with existing literature, which indicates that these muscle groups are most commonly affected by MS-related spasticity and are therefore more frequently treated with BTI (25). BTI dose, number of fESWT impulses and fESWT energy intensity differed significantly across the treated muscle groups, with higher BTI doses, higher fESWT intensity and number of impulses administered in proximal lower limbs. Overall, these findings indicate that treatment intensity (both pharmacological and physical) was modulated according to muscle group. In our population, this reflected a targeted clinical decision-making, that was in line with previous literature, underlining the importance of personalized treatment of BTI dose in MS, influenced by several factors such as disability (26); moreover, also muscle size was described as a factor correlated to BTI dosage (27).

The main limitations of this study are the small sample size and the lack of a control group. Moreover, no comparison with spasticity management in other conditions was performed; therefore, it is not possible to determine whether PwMS may benefit more from this combined approach compared with other conditions, such as stroke or traumatic brain injury. Furthermore, spasticity evaluation was based on the mAS, a scale characterized by high intra-rater reliability but relatively low inter-rater reliability. In most cases, assessments at different time points were performed by the same physician; however, some evaluations were conducted by a different assessor due to clinical practice constraints. Therefore, results derived from the mAS should be interpreted with caution. Finally, the subgroup analyses for each muscle group are based on a very limited number of subjects per group, with a consequent risk of over interpretation of the results.

Despite these limitations, our study provides a preliminary insight into the potential role of combined fESWT and BTI in the management of focal spasticity in MS. This single-session approach may offer both rapid and sustained clinical benefits; however, further studies in larger cohorts and different settings are required to confirm these findings.

## Data Availability

The raw data supporting the conclusions of this article will be made available by the authors, without undue reservation.
