# Supplementary material for: A combined therapeutic approach: extracorporeal shock wave therapy and botulinum toxin in multiple sclerosis-related spasticity
Source: Front Neurol. 2026 Jun 16;17:1851860. doi: 10.3389/fneur.2026.1851860 (PMC13314489; doi:10.3389/fneur.2026.1851860)
Supplement: Supplementary file 1 [file Table_1.DOCX]

Supplementary Material

# Supplementary Tables

Table S1. Estimated marginal means.

| **Muscle group** | **Botulinum toxin dose (U)**  **Mean [95% CI]** | **fESWT hits (Sb)**  **Mean [95% CI]** | **fESWT energy (mJ/mm²)**  **Mean [95% CI]** |
| --- | --- | --- | --- |
| Adductors | 78.75 [64.74, 92.76] | 1021.88 [856.48, 1187.27] | 0.425 [0.391, 0.459] |
| Rectus femoris | 40.77 [25.23, 56.31] | 876.92 [693.44, 1060.41] | 0.431 [0.393, 0.469] |
| Hamstrings | 41.67 [18.79, 64.54] | 1450.00 [1179.91, 1720.09] | 0.400 [0.344, 0.456] |
| Triceps surae | 69.29 [48.11, 90.46] | 1028.57 [778.52, 1278.62] | 0.329 [0.277, 0.380] |
| Biceps brachii | 50.00 [10.38, 89.62] | 750.00 [282.20, 1217.81] | 0.400 [0.304, 0.496] |

Table S2. fESWT and BTI treatment. Continuous variables expressed as mean±SD or median (range), as appropriate.

|  | **Adductors** | **Rectus femoris** | **Hamstrings** | **Triceps surae** |
| --- | --- | --- | --- | --- |
| **N treated patients, n (%)** | 10 (66.7) | 7 (46.7) | 5 (33.3) | 6 (40) |
| **Bilateral treatment, n (%)** | 6 (60) | 6 (85.7) | 1 (20) | 1 (16.7) |
| **BTI dosage** | 80.5±27.3 | 45.0±28.7 | 47.0±33.8 | 72.5±30.1 |
| **fESWT** |  |  |  |  |
| **Hits (Sb)** | 1062 (700-1500) | 750 (700-1500) | 1500 (1400-1500) | 1250 (500-1500) |
| **Energy (mJ/mm^2^)** | 0.40 (0.35-0.50) | 0.40 (0.35-0.50) | 0.40 (0.40-0.40) | 0.40 (0.15-0.40) |
| **Frequency (Hz)** | 4 (4-4) | 4 (4-4) | 4 (4-4) | 4 (4-4) |
| **Pain during fESWT, n (%)** | 0 (0) | 0 (0) | 2 (40%) | 0 (0) |
| **Pain after fESWT, n (%)** | 0 (0) | 0 (0) | 2 (40%) | 0 (0) |
| BTI: botulinum toxin injection; fESWT: focal extracorporeal shockwave treatment. | | | | |

Table S3. mAS, fPSFS, iPSFS, and NRS scores across the four timepoints, expressed as mean±SD or median (range), as appropriate.

|  |  | **T0-pre** | **T0-post** | **T1** | **T2** |
| --- | --- | --- | --- | --- | --- |
| **mAS** | **adductors** | 2.03±0.88 | 1.20±0.92 | 1.30±0.75 | 1.28±0.82 |
|  | **rectus femoris** | 2.50±0.63 | 1.68±0.89 | 1.89±0.92 | 1.96±0.88 |
|  | **hamstrings** | 2.05±1.55 | 1.10±1.02 | 1.20±0.76 | 1.10±0.82 |
|  | **triceps surae** | 2 (0-2) | 1.25 (0-1.50) | 1.25 (0-2) | 0.5 (0-1.5) |
| **fPSFS** | **adductors** | 0 (0-2) | 0 (0-2) | 0 (0-2) | 0 (0-2) |
|  | **rectus femoris** | 0.5 (0-2) | 0.5 (0-2) | 0 (0-2) | 0 (0-2) |
|  | **hamstrings** | 0 (0-3) | 0 (0-3) | 0 (0-2) | 0 (0-1) |
|  | **triceps surae** | 1 (0-3) | 1 (0-3) | 0.25 (0-2) | 0 (0-5) |
| **iPSFS** | **adductors** | 0 (0-2) | 0 (0-2) | 0 (0-1) | 0 (0-1) |
|  | **rectus femoris** | 0.5 (0-2) | 0.5 (0-2) | 0 (0-1) | 0 (0-1) |
|  | **hamstrings** | 0 (0-3) | 0 (0-3) | 0 (0-2) | 0 (0-1) |
|  | **triceps surae** | 0.75 (0-3) | 0.75 (0-3) | 0.25 (0-2) | 0 (0-5) |
| **NRS** | **adductors** | 0 (0-0) | 0 (0-2.5) | 0 (0-5) | 0 (0-1) |
|  | **rectus femoris** | 0 (0-5) | 0 (0-5) | 0.5 (0-5) | 0 (0-5) |
|  | **hamstrings** | 0 (0-0) | 0 (0-0) | 0 (0-5) | 0 (0-7) |
|  | **triceps surae** | 0 (0-6) | 0 (0-1) | 0 (0-0) | 0 (0-0) |

# Supplementary Figures

**Figure S1.** Percentage of muscles with self-reported efficacy of combined treatment, according to timepoints.

**
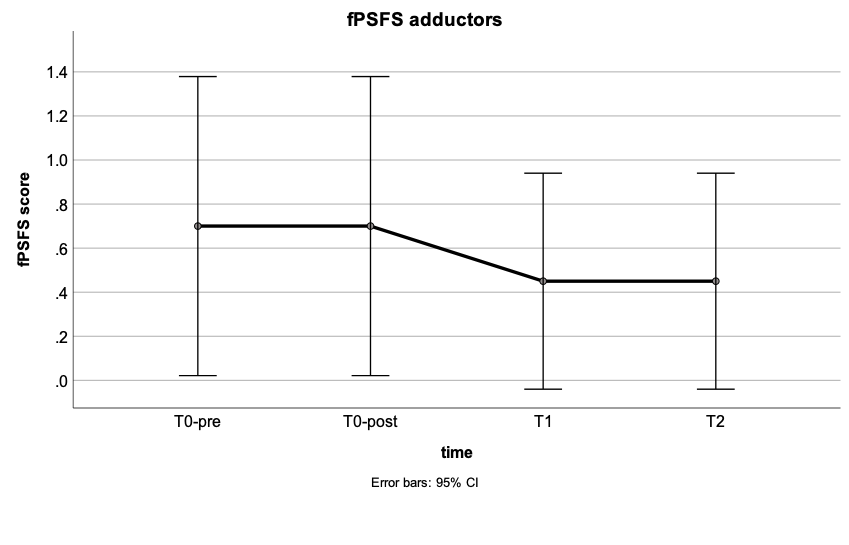

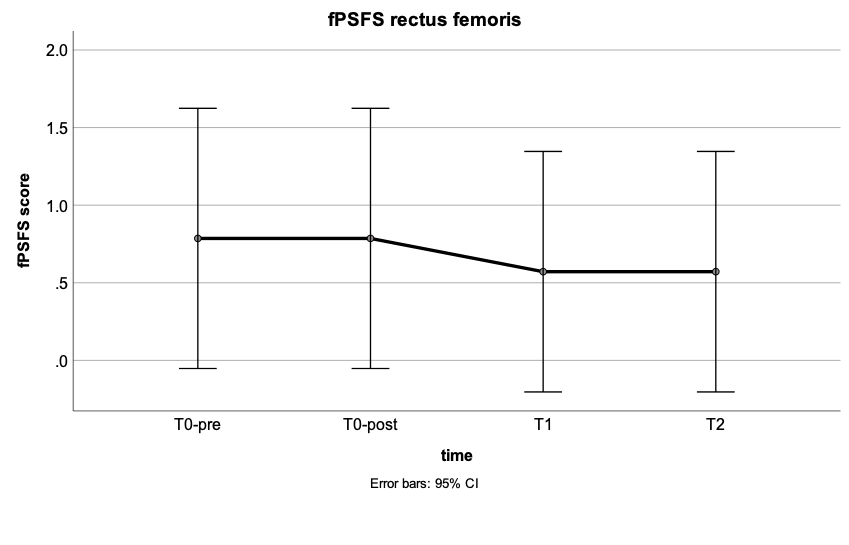
**

**
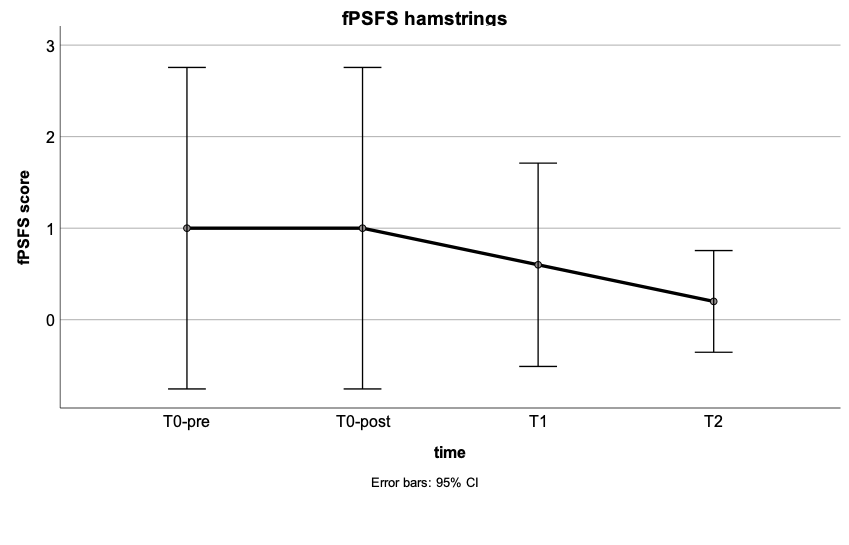

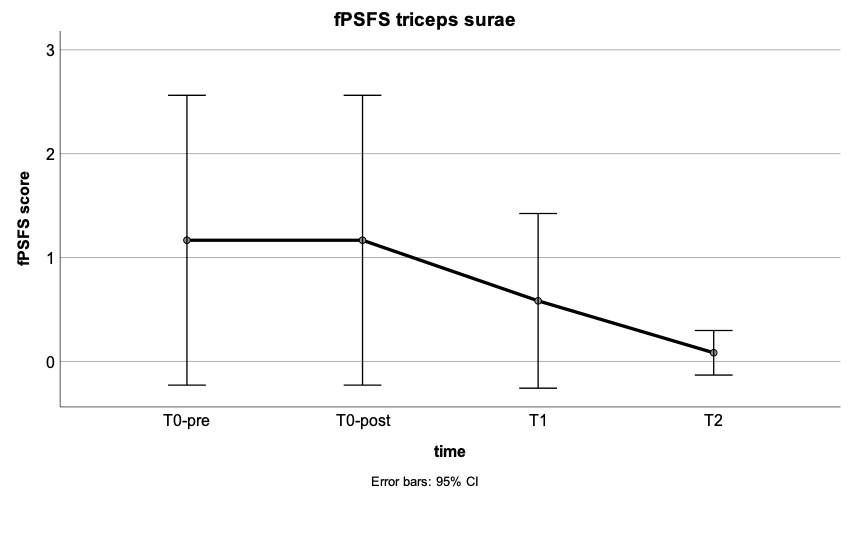
**

**Figure S2.** fPSFS scores in different timepoints.

**
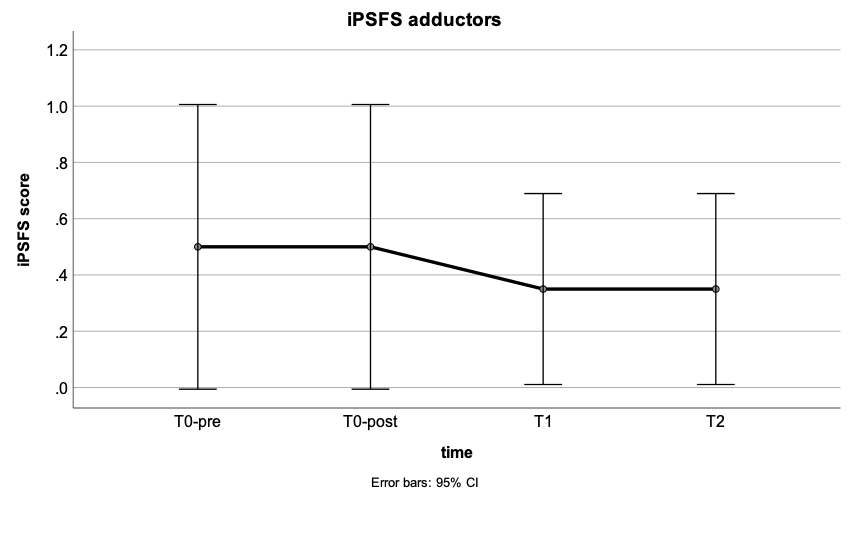

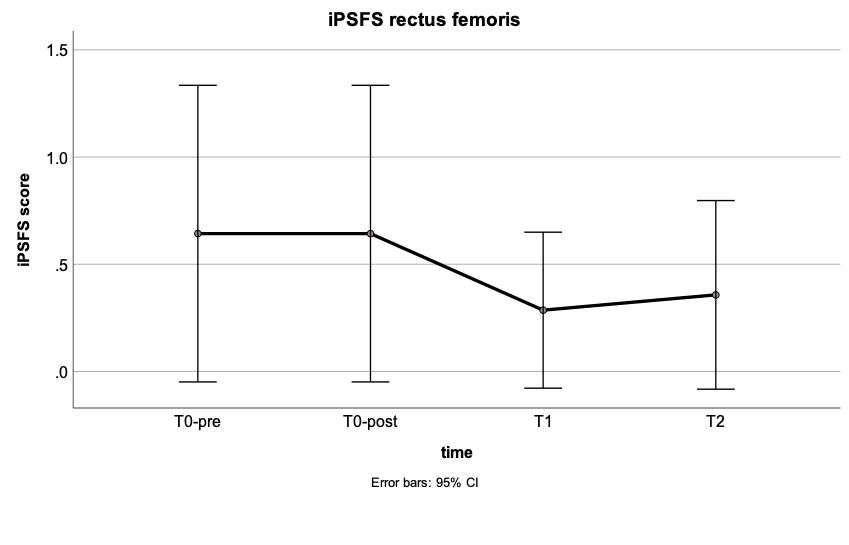
**

**
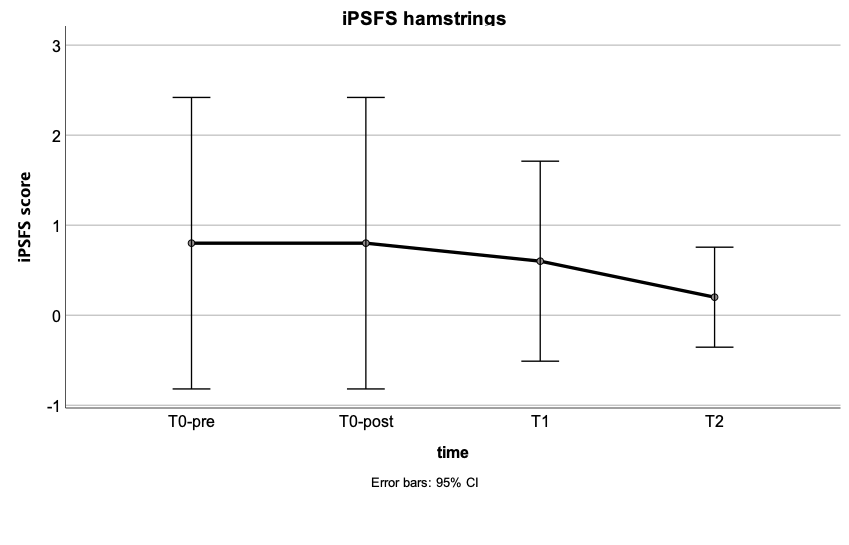

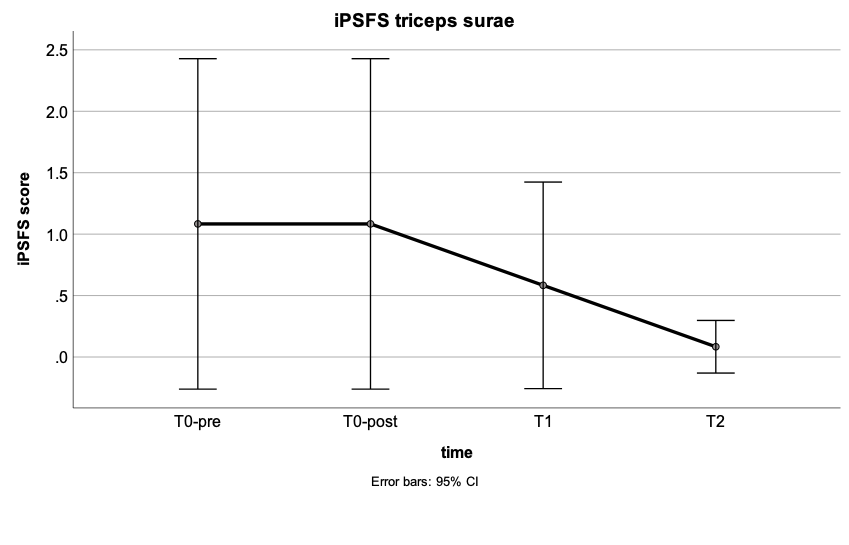
**

**Figure S3.** iPSFS scores in different timepoints.

**
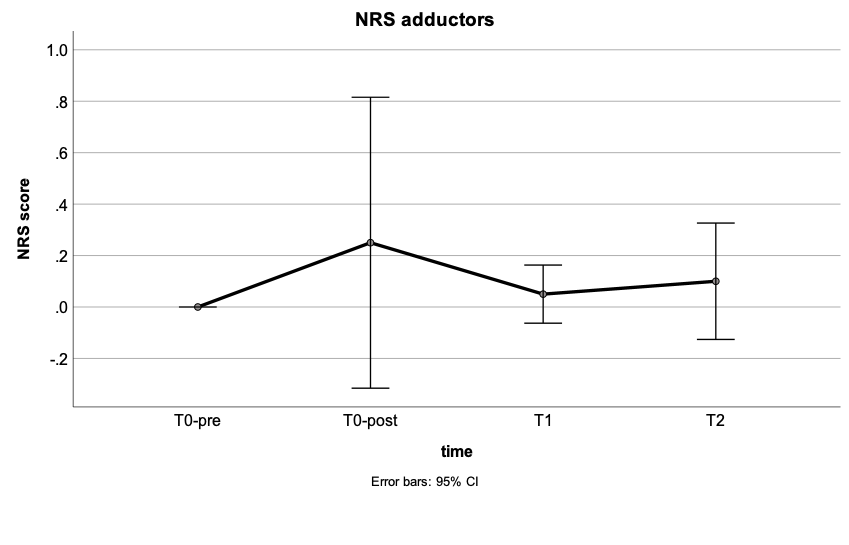

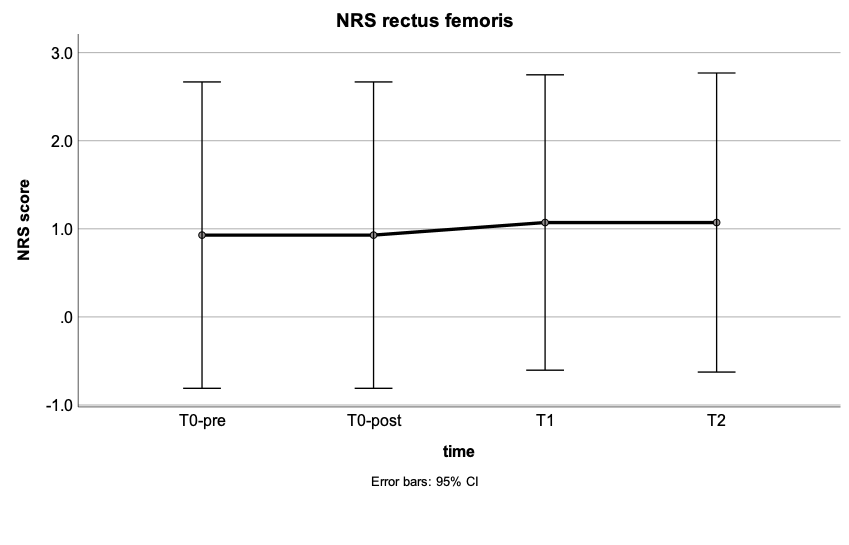
**

**
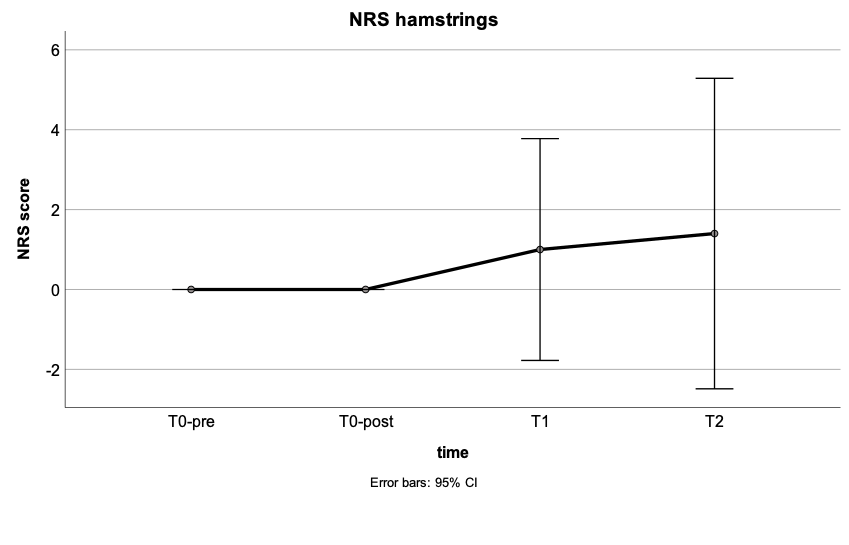

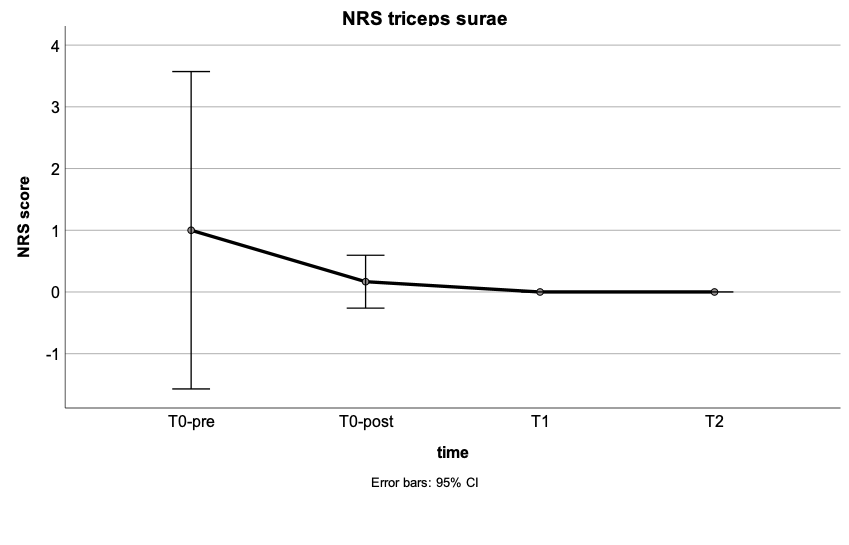
**

**Figure S4.** NRS scores in different timepoints.
